# Within- and between-host dynamics of producer and non-producer pathogens

**DOI:** 10.1017/S0031182023000586

**Published:** 2023-08

**Authors:** Victoria L. Pike, Emily J. Stevens, Ashleigh S. Griffin, Kayla C. King

**Affiliations:** 1 Department of Biology, University of Oxford, Oxford, UK; 2 Department of Zoology, University of British Columbia, Vancouver, Canada; 3 Department of Microbiology & Immunology, University of British Columbia, Vancouver, Canada

**Keywords:** coinfection, pathogen transmission, *Pseudomonas aeruginosa*, quorum sensing, social behaviour

## Abstract

For infections to be maintained in a population, pathogens must compete to colonize hosts and transmit between them. We use an experimental approach to investigate within-and-between host dynamics using the pathogen *Pseudomonas aeruginosa* and the animal host *Caenorhabditis elegans.* Within-host interactions can involve the production of goods that are beneficial to all pathogens in the local environment but susceptible to exploitation by non-producers. We exposed the nematode host to ‘producer’ and two ‘non-producer’ bacterial strains (specifically for siderophore production and quorum sensing), in single infections and coinfections, to investigate within-host colonization. Subsequently, we introduced infected nematodes to pathogen-naive populations to allow natural transmission between hosts. We find that producer pathogens are consistently better at colonizing hosts and transmitting between them than non-producers during coinfection and single infection. Non-producers were poor at colonizing hosts and between-host transmission, even when coinfecting with producers. Understanding pathogen dynamics across these multiple levels will ultimately help us predict and control the spread of infections, as well as contribute to explanations for the persistence of cooperative genotypes in natural populations.

## Graphical abstract

**Figure:**
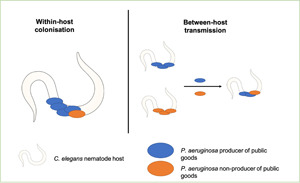

## Introduction

To be successful, pathogens must be able to compete to colonize a host and transmit between new hosts (Anderson and May, 1986). Theory has highlighted the trade-offs that can arise between these two levels as a result of counterbalancing selective pressures within and between hosts (Anderson and May, 1982; Koella and Antia, 1995; Walther and Ewald, 2004). Within the host, pathogens must balance replication and transmission to new hosts (Reece *et al*., 2009), in an analogous trade-off between reproduction and growth within multicellular organisms (Bell, 1980; Clutton-Brock, 1984; Greischar *et al*., 2016). Studying pathogens at these two levels has mainly been theoretical (Anderson and May, 1986; Coombs *et al*., 2007; Mideo *et al*., 2008; Handel and Rohani, 2015), leaving a data gap in disease ecology (Coombs *et al*., 2007; Mideo *et al*., 2008; Handel and Rohani, 2015). Understanding this interplay is crucial for understanding the fitness and transmissibility of pathogens, which could help contribute to developing effective intervention strategies (Hellriegel, 2001; Handel and Rohani, 2015) for clinically important pathogens, such as *Plasmodium sp.* (Greischar *et al*., 2016) to those having major effects on ecosystems, such as the fungi *Batrachochytrium dendrobatidis* (Pedersen *et al*., 2007; Voyles *et al*., 2009; Kilpatrick *et al*., 2010; Wilber *et al*., 2017).

A pathogen rarely infects a host alone (Petney and Andrews, 1998; Pedersen and Fenton, 2007; Rynkiewicz *et al*., 2015; Betts *et al*., 2016). The presence of coinfecting strains may alter the within-host dynamics of an individual pathogen (Mideo, 2009). For example, in the host, there may be competition for resources (Pedersen and Fenton, 2007; Mideo, 2009), including key nutrients such as iron (Ratledge and Dover, 2000; Nairz *et al*., 2010; Kramer *et al*., 2019). These within-host interactions may in turn alter between-host dynamics, as the presence of competitors may mean host exploitation is optimal (Frank, 1996; Alizon *et al*., 2013) with reduced selection for transmission to secure a larger proportion of resources within the host (McKenzie and Bossert, 1998; Greischar *et al*., 2016).

Within-host interactions are of vital importance for understanding infection outcomes (Foster, 2005; West *et al*., 2007; Leggett *et al*., 2014; Rezzoagli *et al*., 2020). It has been well established that pathogens can interact cooperatively to successfully colonize a host, for example, *via* forming biofilms (Griffin *et al*., 2004; Kreft, 2004; Foster, 2005; Diggle *et al*., 2007*b*; West *et al*., 2007). Social traits such as the production of public goods into the local environment (West *et al*., 2006, 2007; Leggett *et al*., 2014) are frequently involved in these pathogen interactions. Public goods benefit all the cells in the local environment, but they are costly to produce and thus are susceptible to exploitation by pathogens which may utilize but not produce public goods (non-producers) (Velicer, 2003; West *et al*., 2007). For example, the production of compounds known as siderophores that are used to bind to and uptake iron (Buckling *et al*., 2007; Kramer *et al*., 2019), benefitting all bacteria in the local area as iron, is often limited within a host (Ratledge and Dover, 2000; Nairz *et al*., 2010). The release of products such as siderophores is regulated by communication between pathogens using small autoinducer molecules in a process known as quorum sensing (QS) (Keller and Surette, 2006; West *et al*., 2007; Williams *et al*., 2007; Rumbaugh *et al*., 2009). Non-producers for both siderophore production and QS have been shown to arise in infections (De Vos *et al*., 2001; Köhler *et al*., 2009; Jiricny *et al*., 2014; Andersen *et al*., 2015). Yet when populations are sampled at random, non-producers are rare (Köhler *et al*., 2009; Andersen *et al*., 2015), suggesting that non-producers could be poor at between-host transmission even if they are effective at invading established infections. Non-producers have been shown to be able to invade *in vitro* (Griffin *et al*., 2004; Diggle *et al*., 2007*b*) and *in vivo* (Rumbaugh *et al*., 2009). Compared to our understanding of how pathogens interact within a host, relatively little is known about how within-host processes contribute to variation in pathogen transmission between hosts (Brown *et al*., 2009; Handel and Rohani, 2015; VanderWaal and Ezenwa, 2016; Stephenson *et al*., 2017).

In this study, we use the well-established model bacterial pathogen, *Pseudomonas aeruginosa* (Griffin *et al*., 2004; Buckling *et al*., 2007; Diggle *et al*., 2007*b*) to investigate the role of public goods production on within-host colonization and between-host transmission. Each of the two non-producer strains we used was unable to produce a public good; one strain did not produce a type of siderophore, and the other was not able to quorum sense. Both of these traits are well-established for investigating cooperative interactions between bacterial cells (Griffin *et al*., 2004) with consequences for virulence in the case of QS (Rumbaugh *et al*., 2009). We predicted that producer (potentially cooperative) strains would be better able to transmit even if they were outcompeted by exploitative non-producers (potential cheats). Investigating pathogen transmission between hosts can be logistically challenging as it requires large population sizes (Handel and Rohani, 2015). We thus used the model *Caenorhabditis elegans* as the host, enabling us to have large populations with natural between-host fecal–oral transmission (Kenney *et al*., 2005; Diaz and Restif, 2014). We began by tracking the within-host dynamics of producers and non-producers, in single infections and coinfections, over time. We varied the order of exposure of the different types of pathogen to determine whether priority effects altered within-host interactions (Goodman and Ross, 1974; de Roode *et al*., 2005; Jackson *et al*., 2006; Clay *et al*., 2018). We then introduced infected nematodes into uninfected populations and measured rates of between-host transmission. We investigated whether differences in pathogen strain transmissibility were due to variation in host preference for ingesting a particular pathogen strain or in host shedding into the environment. Overall, our study demonstrates that, in contrast to expectations from *in vitro* competition assays (Griffin *et al*., 2004; Diggle *et al*., 2007*b*; Kümmerli *et al*., 2009), producer pathogens are superior to non-producers at within-host colonization and between-host transmission in this species interaction.

## Materials and methods

### Bacterial pathogen and nematode host

*Caenorhabditis elegans* is a nematode species whose natural diet is composed of microorganisms (Hope, 1999). The gut of *C. elegans* can be colonized by a variety of microbes, including pathogens (Clark and Hodgkin, 2014). We used 2 strains of *C. elegans* N2 and CB5584 (*mIs12 II*). CB5584 express fluorescence in their pharynx making them distinguishable from N2 under a fluorescent microscope. We selected CB5584 as it enabled us to identify nematodes from different populations, those exposed to pathogens (N2) and pathogen-naive populations (CB5584) without any difference in pathogen susceptibility (Wang, 2020). Nematodes were maintained at 20°C on a lawn of food [*Escherichia coli* (OP50)] on nematode growth medium (NGM) plates. To synchronize life cycles, nematodes were treated with bleach (NaClO and sodium hydroxide) which kills everything except unhatched eggs (Hope, 1999). After bleaching, nematodes were synchronized overnight in M9 buffer and maintained for 2 days.

We exposed nematodes to the Gram-negative pathogen, *P. aeruginosa*, an opportunistic pathogen of plants and animals, including humans (Tan *et al*., 1999). On our selected media, *P. aeruginosa* is a slow killing pathogen of *C. elegans* (Tan *et al*., 1999). We used 3 strains of *P. aeruginosa* in our experiments with the same genetic background (PAO1 strain background). The ‘producer’ strain was: PAO1 WT::GFP (WT labelled with GFP, which we herein refer to as the producer) and 2 ‘non-producer’ strains: PAO1 ΔlasR::mCherry (QS non-producer labelled with mCherry, which we herein refer to as non-producer A); and PAO1 Δpvd::mCherry [siderophore (pyoverdine) non-producer strain labelled with mCherry, which we herein refer to as non-producer B] (Rezzoagli *et al*., 2019). The non-producer strains differed. Non-producer A lacked the ability to communicate with other bacteria *via* QS (Keller and Surette, 2006; West *et al*., 2007; Williams *et al*., 2007; Rumbaugh *et al*., 2009) which also affects a variety of other traits (e.g. biofilm development; Williams *et al*., 2007; Diggle *et al*., 2007*b*). Comparatively, non-producer B had only lacked the ability to produce the single siderophore pyoverdine (Buckling *et al*., 2007; Visca *et al*., 2007). Each strain had a visually distinct colony morphology which enabled colony counting in the 2-stage exposure experiment.

For our investigation of nematode preference, we also used 2 strains of *Pseudomonas fluorescens*: a producer (CHA0) and a non-producer (CHA019) of *gacS* defensive toxins (Jousset *et al*., 2009). *Caenorhabditis elegans* have previously been shown to preferentially graze on the non-producer, CHA019 (Jousset *et al*., 2009), and so we used it as a positive control. All bacteria were stored at −80°C in a 1:1 ratio of sample to 50% glycerol solution in cryotubes. Strains of *Pseudomonas* bacteria were grown overnight at 37°C in Lysogeny broth shaking at 200 r.p.m. OP50 was grown under the same conditions at 30°C.

### Within-host colonization and dynamics

#### Part 1 (I): single exposure

Approximately 1000 nematodes at the L4 stage were transferred onto a lawn of either the producer, non-producer A, or non-producer B for 24 h. The plates were saturated with bacteria such that the nematodes had a continuous grazing source. For the first stage of the experiment, a sample of 4–5 nematodes were removed from the single pathogen treatment after 24 h and washed according to the droplet method (Ford *et al*., 2016) (Fig. S1). Treatments for this experiment consisted of five biological replicates, and the whole experiment was replicated four times.

#### Part 1 (II): 2-stage exposure

The two-pathogen treatment involved the pathogen strain being switched after 12 h exposure, with an additional washing stage between transfers (see Fig. S1). We exposed nematodes to the pathogen treatments sequentially. Simultaneous exposure would have resulted in competition outside of the host, which we wanted to avoid. There were 4 different treatments according to the order of primary and secondary pathogen exposure: (i) producer to non-producer A, (ii) producer to non-producer B, (iii) non-producer A to producer and (iv) non-producer B to producer. This exposure method allowed us to investigate whether the order of exposure affected within-host interactions.

To obtain an estimate of pathogen load, we calculated the number of colony-forming units (CFUs) per nematode. We placed 4–5 clean nematodes in 90 *μ*L of M9 in a 1 ml Eppendorf tube (containing microbeads). The tube was placed in a bead-beater for 1 min at 2800 rpm. After crushing, serial dilutions were plated onto Kings Broth (KB) media (Ghoul *et al*., 2016) and grown overnight at 30°C. The number of colonies was counted. Treatments for both stages of the experiment consisted of 5 biological replicates, and the whole experiment was replicated four times.

For details of an additional 2-stage exposure analysis, see Supplementary materials. In this analysis, we conduct time controls using the nematode food (OP50) and demonstrate that the time of exposure (i.e. 0 h or after 12 h) does not affect colonization ability of any of the pathogen types.

### Between-host dynamics

#### Part 2: between-host transmission

To investigate between-host dynamics, we used nematodes from the within-host assay (see Fig. S1). After the nematodes' exposure in the first instance to a single pathogen (either non-producer or producer), and in the second stage exposure to a two-pathogen treatment, a sample of ten clean nematodes were transferred to a pathogen-naive population of GFP-labelled nematodes (see Fig. S1, part 2). After 24 h, we took 4–5 pathogen-naive nematodes from each plate to calculate pathogen load by counting the CFUs within nematodes. We were able to distinguish, and selectively pick, the pathogen-naive nematodes using a fluorescent microscope. The pathogen-naive worms were distinguished by their green pharynx.

#### Preference assay

To determine whether nematodes preferred to consume producers or non-producers, we conducted a preference assay. Bacterial cell density was measured at an absorbance of 600 nm (A_600_) and then standardized to a density of 1 using M9. We used these standardized cultures to make equidistant 20 *μ*L spots on a 90 mm KB media (Ghoul *et al*., 2016) plate (see Fig. S3A). Each KB plate contained 1 spot of producer and a spot of either non-producer A, non-producer B or the control *P. fluorescens* (CHA0 or CHA019). We left the bacteria to dry and then incubated the plates overnight at 30°C . We introduced approximately 40 clean nematodes to the centre of to the prepared plates with equidistant producer and an alternative pathogen (non-producer A, non-producer B, CHA019 or CHA0) [as in Abada *et al*. (2009)]. We recorded the approximate time of bacteria drying as 0 h and left the plates for 6 h [by this time nematodes are likely to have established their preference (Shtonda and Avery, 2006; Ballestriero *et al*., 2016)]. Treatments consisted of six biological replicates and the whole experiment was replicated five times. After 6 h, we recorded the number of nematodes on the producer, non-producer, or neither colony.

#### Host shedding of pathogens into the environment assay

To investigate how many pathogens were shed from the nematode host into the environment, we exposed nematodes to either a producer or non-producer *P. aeruginosa* (non-producer A or B) for 12 h. These exposed nematodes were cleaned and transferred to an empty NGM plate (with a minimal lawn of OP50). After 12 h, these nematodes were picked off the plate and the pathogen cells liberated using M9 buffer. The bacteria from the plate were then grown and counted for comparison (Fig. S3B). Pathogen colonies were phenotypically distinguishable from OP50.

### Statistical analysis

All of the statistical analysis and data presentation was carried out in R version 3.6.2 (R Core Team, 2019) using RStudio (RStudio Team, 2021) and the packages ‘dplyr’ (Wickham *et al*., 2017), ‘ggplot2’ (Wickham, 2009), ‘multcomp’ (Hothorn *et al*., 2008), ‘cowplot’ (Wilke, 2020), ‘stargazer’ (Hlavac, 2018), ‘broom’ (Robinson *et al*., 2021), ‘lme4’ (Bates *et al*., 2015) and ‘lmerTest’ (Kuznetsova *et al*., 2017). Results were considered to be statistically significant when *P* < 0.05.

#### Within-host colonization and dynamics part 1 (I): single exposure

We compared pathogen loads within hosts exposed to either a non-producer or producer. We fit a linear model with ‘CFU per nematode’ as the response variable and ‘pathogen’ [3 treatments: (i) producer, (ii) non-producer A and (iii) non-producer B] as the explanatory variable. We then carried out an ANOVA with this model and ran post-hoc Tukey tests.

#### Within-host colonization and dynamics part 1 (II): 2-stage exposure

When nematodes were exposed to both a non-producer and producer [4 ‘pathogen’ treatments, written in order of exposure: (i) producer to non-producer A, (ii) producer to non-producer B, (iii) non-producer A to producer and (iv) non-producer B to producer], we fit a quasi-Poisson generalized linear model (GLM) to compare pathogen loads (with either ‘non-producer CFU per nematode’ or ‘producer CFU per nematode’ as the response variable and ‘pathogen’ as the explanatory variable). To assess the significance of ‘pathogen’, we carried out a likelihood ratio test with this model and a second model from which the ‘pathogen’ term had been dropped. We then ran post-hoc Tukey tests using the ‘glht()’ function from the multcomp package (Hothorn *et al*., 2008) to assess pairwise differences.

#### Between-host dynamics part 2: between-host transmission

To investigate between-host dynamics within the pathogen-naive nematode populations (introduced to nematodes that had been singly infected with either a producer or a non-producer), we compared pathogen loads across treatments. We fit a linear model with ‘CFU per nematode’ as the response variable and ‘pathogen’ [3 treatments: (i) producer, (ii) non-producer A and (iii) non-producer B] as the explanatory variable. We carried out an ANOVA to calculate *F* ratios for the explanatory variable ‘pathogen’ and ran post-hoc Tukey tests. To compare the pathogen loads from the pathogen-naive populations, where the introduced infected nematodes had been exposed to both producers and non-producers [4 ‘pathogen’ treatments, written in order of exposure: (i) producer to non-producer A, (ii) producer to non-producer B, (iii) non-producer A to producer and (iv) non-producer B to producer], we fit a quasi-Poisson GLM. For the GLM, the response variable was either the pathogen load of non-producers (‘non-producer CFU per nematode’) or that of the producer (‘producer CFU per nematode’) and ‘pathogen’ as the explanatory variable. To assess the significance of the explanatory variable ‘pathogen’, we carried out a likelihood ratio test with this model and a second model from which the ‘pathogen’ term had been dropped. We then ran post-hoc Tukey tests.

#### Preference assay and host shedding of pathogens into the environment assay

To investigate whether nematodes displayed a preference for either producers or non-producers, we calculated a choice index: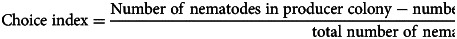 where a value of 1 indicates complete preference for the producer, 0 indicates a lack of preference and −1 indicates complete preference for the alternative strain (all non-producers except for CHA0). We tested whether the choice indexes were significantly different from zero (indicating no preference) using 1-sample *t*-tests. Finally, to compare the number of CFUs present on the lawn of a plate after an infected population had been removed, we fit a quasi-Poisson GLM with ‘CFU per nematode’ as the response variable and ‘pathogen’ as the explanatory variable. We conducted a likelihood ratio test with this model and a second model from which the ‘pathogen’ term had been dropped and ran post-hoc Tukey tests between the pathogen treatments.

## Results

### Within-host dynamics: producers are better than non-producers at colonizing nematode hosts

#### Part 1 (I): single exposure

**Figure 1.**
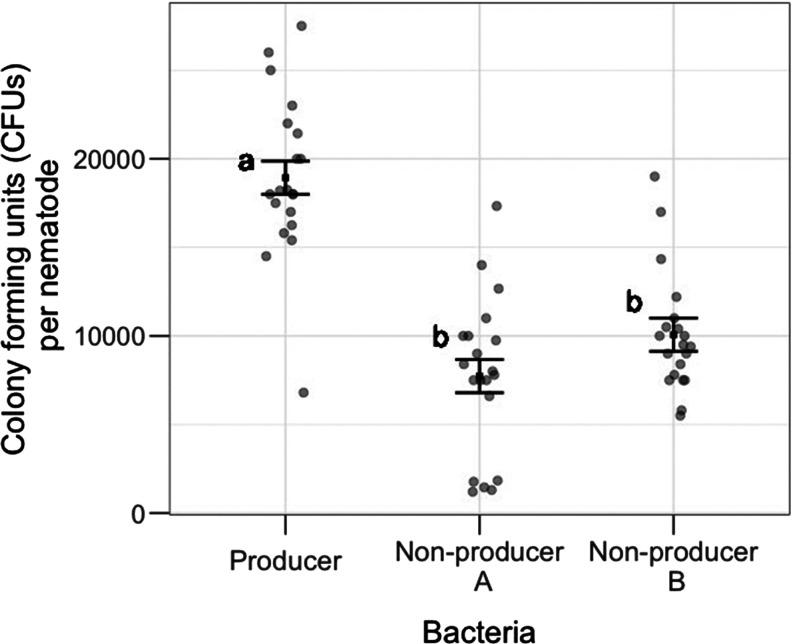
Within-host colonization. Difference in the pathogen load, measured by the number of colony-forming units (CFUs) per nematode, after exposure to one of the three strains of *P. aeruginosa.* Treatments with different letters are significantly different. Square points with error bars represent mean ±1 s.e.

The producer was better able to colonize the nematode host than both non-producers, as the pathogen load (CFUs per nematode) was significantly higher for producers than non-producer A or B (post-hoc Tukey tests; non-producer A–producer: mean difference = −11 200, *P* < 0.001; non-producer B–producer: mean difference = −8870, *P* < 0.01, see Fig. 1, Table S1). There was no difference between the pathogen loads for nematodes exposed to non-producer A or B (post-hoc Tukey test; non-producer B–non-producer A: mean difference = −2336, *P* = 0.19, Fig. 1, Table S1), but overall, there was a significant effect of ‘pathogen’ treatment (*F*_2,57_ = 39.8, *P* < 0.001, Table S1, Fig. 1).

#### Part 1 (II): 2-stage exposure

**Figure 2.**
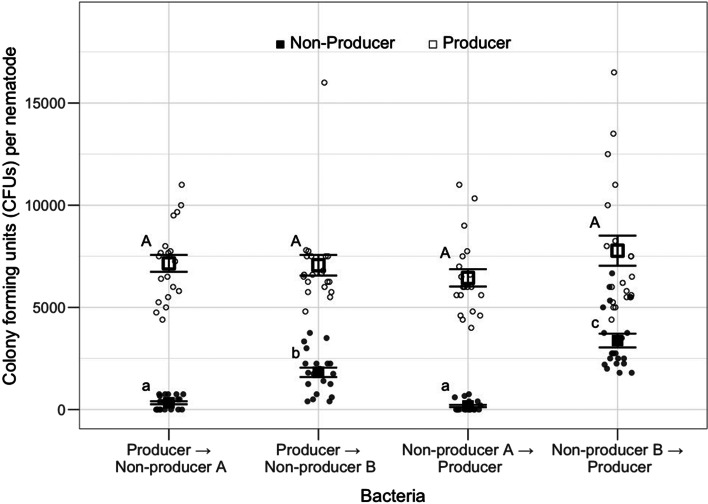
Within-host colonization and dynamics of co-infecting producer and non-producer pathogens. Difference in the producer (white) and non-producer (black) pathogen loads, measured by the number of colony-forming units (CFU) per nematode, after co-exposure to two pathogens (producer and non-producer A or non-producer B). The *x*-axis labels are given in order of exposure. Points with different letters are significantly different from each other. Square points with error bars represent mean ±1 s.e.

When nematodes were exposed to both producers and non-producers, producers colonized nematodes at a similar level across pathogen treatments (no effect of treatment: likelihood ratio: deviance = 2500, *P* = 0.365, Fig. 2, Table S2B). There was a significant effect of pathogen treatment for non-producer CFUs (deviance = 100 414, *P* < 0.001, Fig. 2, Table S2A). The pathogen load of both non-producer B treatments (producer to non-producer B and non-producer B to producer) was significantly higher than both non-producer A treatments (producer to non-producer A and non-producer A to producer), regardless of the order of exposure, with non-producer B to producer having a significantly higher number of CFUs per nematode than all the other treatments (Fig. 2, Table S2A).

### Between-host dynamics: producers are better at between-host transmission than non-producers

#### Part 2: between-host transmission

**Figure 3.**
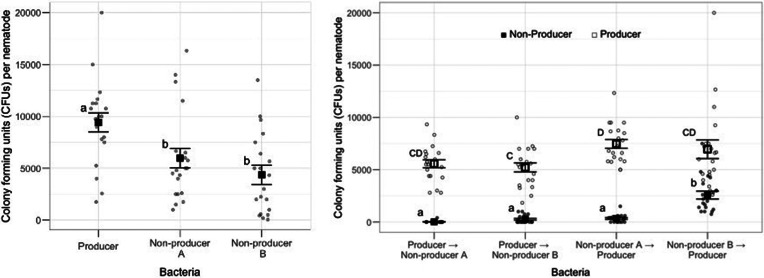
Between-host dynamics of producer and non-producer pathogens. (A) Difference in the pathogen load (CFUs per nematode) from the naive population after singly-infected nematodes were introduced. (B) Difference in the number of producers (white) and non-producers (black) in the colony-forming units (per nematode), from the naive population after co-infected nematodes were introduced. The *x*-axis labels are given in order of exposure. Points with different letters are statistically significantly different. Square points with error bars represent mean ±1 s.e.

When nematodes infected by asingle pathogen strain were introduced to the naive population, the producer pathogens spread best (*F*_2,57_ = 7.73, *P* = 0.001, post-hoc Tukey tests; producer to non-producer A: mean difference = −3440, *P* = 0.030; producer to non-producer B: mean difference = −5040, *P* = 0.0009, see Fig. 3A, Table S5).

When nematodes were co-infected by producers and non-producers, the pathogen load of producer in the pathogen-naive populations of nematodes remained consistently high (Fig. 3B). Although pathogen loads were similar across treatments, there was a significant effect of ‘pathogen’ (likelihood ratio test: ‘producer’ deviance = 11 100, *P* = 0.01, Table S6B) likely driven by the statistically significant difference between the loads of the producer to non-producer B and non-producer A to producer treatments (Tukey post-hoc test of difference: mean of ‘non-producer A to producer’–‘producer to non-producer B’ = 0.358, *P* = 0.026). The non-producer pathogen loads of nematodes were low and close to zero across treatments (Fig. 3B), except for the non-producer B to producer treatment where there was a significantly higher number of CFUs per nematodes than all the other types of non-producer (Table S6). This higher number of CFUs per nematode for non-producer B to producer treatment resulted in a significant effect of ‘pathogen’ (likelihood ratio test: ‘non-producer’ deviance = 94 200, *P* < 0.001).

#### Preference assay

**Figure 4.**
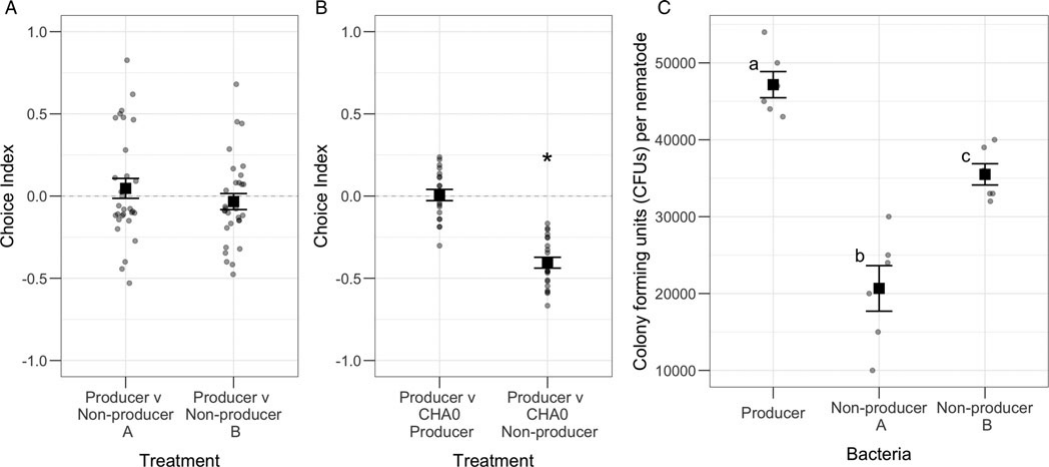
Host preference for picking-up pathogens and host shedding of pathogens in the environment. (A) and (B) Choice index of *C. elegans* for producer and non-producer strains of *P. aeruginosa*. Where 1 indicates complete preference for the producer, 0 indicates a lack of preference for either and −1 indicates complete preference for the alternative strain (non-producer A, non-producer B, CHAO19 and CHA0). Figure (A) shows the choice index for the two focal non-producer strains non-producer A and non-producer B in comparison to the producer whereas (B) shows the choice index for *P. fluorescens* in comparison to the producer. * Indicates significance. (C) Difference in the number of colony-forming units (CFUs) present on a lawn after 12 h of exposure to infected nematodes. Each of the 3 points is statistically significantly different from each other denoted by different letters. Square points with error bars represent mean ±1 s.e.

*Caenorhabditis elegans* did not display a preference for either the non-producer or producer strains of *P. aeruginosa* (see Fig. 4A, Table S7A). The choice index was not significantly different from zero for either producer *vs* non-producer A (mean choice index = 0.0468, *t* = 0.733, *P* = 0.45) or producer *vs* non-producer B (mean choice index = −0.0333, *t* = −0.683, *P* = 0.5). As a positive control, we found *C. elegans* showed a preference for the non-producer *P. fluorescens* strain CHA019 (mean choice index = −0.405, *t* = −12.28, *P* < 0.001, Fig. 4B) as in Jousset *et al*. (2009). There was no preference for the producer *P. protegens* strain CHA0 (mean choice index = 0.00641, *t* = 0.188, *P* = 0.85, Fig. 4B).

#### Host shedding of pathogens into the environment assay

Nematodes shed significantly more producers into the environment than either type of non-producer (overall effect of ‘pathogen’ likelihood ratio test: deviance = 64 100, *P* < 0.001; post-hoc Tukey tests; producer–non-producer A: difference = −0.825, *P* < 0.001; non-producer B–producer: difference = −0.284, *P* = 0.007, Fig. 4C, Table S7B). There was also a significant difference between the number of CFUs present on a lawn between the two non-producer strains. More non-producer B pathogens were shed into the environment than non-producer A (non-producer B–non-producer A = 0.541, *P* < 0.001, Fig. 4C, Table S7B).

## Discussion

We revealed that public-good producers showed superior ability to establish infection within- and between-nematode hosts compared to non-producers. There were consistently more producers infecting nematodes when the hosts were directly exposed to the pathogens as well as when pathogens were able to spread naturally between hosts. Non-producers were poor at both within-host colonization and between-host transmission, even when coinfecting hosts with producers. Our results suggest that a higher pathogen load for producers is related to an increase in their ability to transmit between hosts. This positive relationship between pathogen load and transmissibility is a common assumption in theoretical studies (see Handel and Rohani, 2015 and references therein), but with few empirical examples (guppy [*Poecilia reticulata*] ectoparasite [*Gyrodactylus turnbulli*] system in Stephenson *et al*., 2017).

The counterbalancing selection pressures experienced by pathogens at the level of within-host colonization and between-host transmission can lead to trade-offs (Anderson and May, 1982; Koella and Antia, 1995; Walther and Ewald, 2004). Within a host, the ability to reproduce rapidly may be advantageous to outcompete competitors. However, pathogens that reproduce more slowly may be able to persist for longer periods in the external environment. This ability could be a beneficial trait for transmission, but disadvantageous for colonization (Walther and Ewald, 2004). These trade-offs can be affected by multi-species interactions within a host (Mideo, 2009; Alizon *et al*., 2013), and by transmission mode (Walther and Ewald, 2004; Antonovics *et al*., 2017). Counter to our expectations, we do not find evidence for an existing trade-off between within-host colonization and transmissibility for producer pathogens.

We predicted that in the absence of producers, non-producers would be less able to colonize the host. Non-producers lack the ability to either produce iron-scavenging siderophores (non-producer B [pyoverdine]) (Buckling *et al*., 2007) or induce the las QS pathway (non-producer A) (Keller and Surette, 2006; West *et al*., 2007; Diggle *et al*., 2007*b*, 2007*a*). Non-producer A was relatively poor at colonizing and transmitting between hosts compared to non-producer B, an outcome likely due to the more severe fitness consequences experienced by non-producer A than non-producer B. Non-producer A is unable to communicate with other pathogens *via* QS (Keller and Surette, 2006; Venturi, 2006). This is a crucial trait to the success of *P. aeruginosa* that controls behaviours, such as biofilm development and the production of virulence factors (Williams *et al*., 2007; Diggle *et al*., 2007*b*). While non-producer B does not produce the primary siderophore pyoverdine (Buckling *et al*., 2007; Visca *et al*., 2007), which can negatively impact the fitness of the strain in an iron-limited host environment (Ratledge and Dover, 2000; Nairz *et al*., 2010), it can produce other siderophores and can take up iron from the environment in other ways (Buckling *et al*., 2007; Visca *et al*., 2007). In addition, nematode hosts did not preferentially consume pathogenic producers over non-producers. There was no difference in the intake of pathogen type. More producers, however, were shed into the local environment than non-producers, making them more successful at between-host transmission than non-producers.

Even in the presence of producers, non-producers were less able to colonize hosts and transmit between them. This inability of non-producer strains to exploit producers (i.e. their inability to cheat effectively) has previously been found in *in vivo* studies using nematode hosts (Rezzoagli *et al*., 2019), as well as in larvae of the wax moth (*Galleria mellonella*) (Harrison *et al*., 2006) and the diamondback moth (*Plutella xylostella*) (Zhou *et al*., 2014). We expect that this outcome is likely due to several factors. Firstly, within the nematode gut, there is a relatively low pathogen density (Rezzoagli *et al*., 2019). Non-producers are most likely to benefit from producers when they exist at a low density (Ross-Gillespie *et al*., 2007; Rumbaugh *et al*., 2009) but within a high density of producers (Ross-Gillespie *et al*., 2009), in a negative-frequency dependent interaction. Secondly, the spatial heterogeneity within the host may not be conducive to cheating (Harrison *et al*., 2006; Leggett *et al*., 2014; Rezzoagli *et al*., 2019). The *in vitro* environment is more homogenous (Pedersen and Fenton, 2007; Mideo, 2009), allowing non-producers to mix with producers and potentially utilize the products released into the environment (Griffin *et al*., 2004; Diggle *et al*., 2007*b*; Kümmerli *et al*., 2009). Whereas *in vivo*, the environment is likely to have more structure, preventing mixing. Therefore, within the host, non-producers may be more likely to be surrounded by their non-producer relatives (Frank, 1998), and thus will be less able to directly benefit from the public goods of the producers (Harrison *et al*., 2006; West *et al*., 2006; Rezzoagli *et al*., 2019). Finally, the nematode host itself may impact the non-producer's colonization ability; limited iron availability may remove any benefit of not producing the costly siderophores (Ratledge and Dover, 2000; Nairz *et al*., 2010).

Understanding how social traits affect within- and between-host dynamics requires further experiments (Mideo *et al*., 2008; Handel and Rohani, 2015). Our study, among others (Diaz and Restif, 2014), highlights the benefits of *C. elegans* as a model host for studying between-host dynamics without major logistical challenges (Handel and Rohani, 2015). A future avenue for exploration within this system could involve experimentally evolving non-producers *de novo* within the nematode that can successfully cheat (Ghoul *et al*., 2014) to determine how within-host cheating impacts pathogen transmissibility. Cheats have been shown to evolve in natural populations, for example, QS cheats are found in the lungs of cystic fibrosis patients (Diggle *et al*., 2007*a*, 2007*b*). Successful within-host cheating could negatively impact the transmission dynamics of an infection and could be explored as a potential disease intervention strategy (Brown *et al*., 2009; Jiricny *et al*., 2014; Leggett *et al*., 2014).

We suggest that within-host pathogen interactions affect between-host infection dynamics. We provide empirical evidence linking processes occurring at different scales, in a field dominated by theory (Coombs *et al*., 2007; Mideo *et al*., 2008; Handel and Rohani, 2015), investigating the role of pathogenic social traits important in disease. Taking a multilevel experimental approach to within- and between-host dynamics is an outstanding and major challenge within evolutionary biology (Mideo *et al*., 2008). Addressing this challenge will improve our ability to predict and control infection in less well-understood systems, such as disease-induced population extinctions (Pedersen *et al*., 2007; Smith *et al*., 2009; Wilber *et al*., 2017) and may also allow us to harness pathogen interactions for our own benefit (Smith and Holt, 1996; Mideo, 2009; Leggett *et al*., 2014; Rezzoagli *et al*., 2020).

## Data Availability

The data and code from this study have been uploaded with the supplementary material.

## Supplementary material

For supplementary material accompanying this paper visit https://doi.org/10.1017/S0031182023000586. click here to view supplementary material
