# Supplementary material for: Within- and between-host dynamics of producer and non-producer pathogens
Source: Parasitology. 2023 Jul 3;150(9):805–12. doi: 10.1017/S0031182023000586 (PMC10478067; doi:10.1017/S0031182023000586)
Supplement: Supplementary file 1 [file S0031182023000586sup001.pdf]

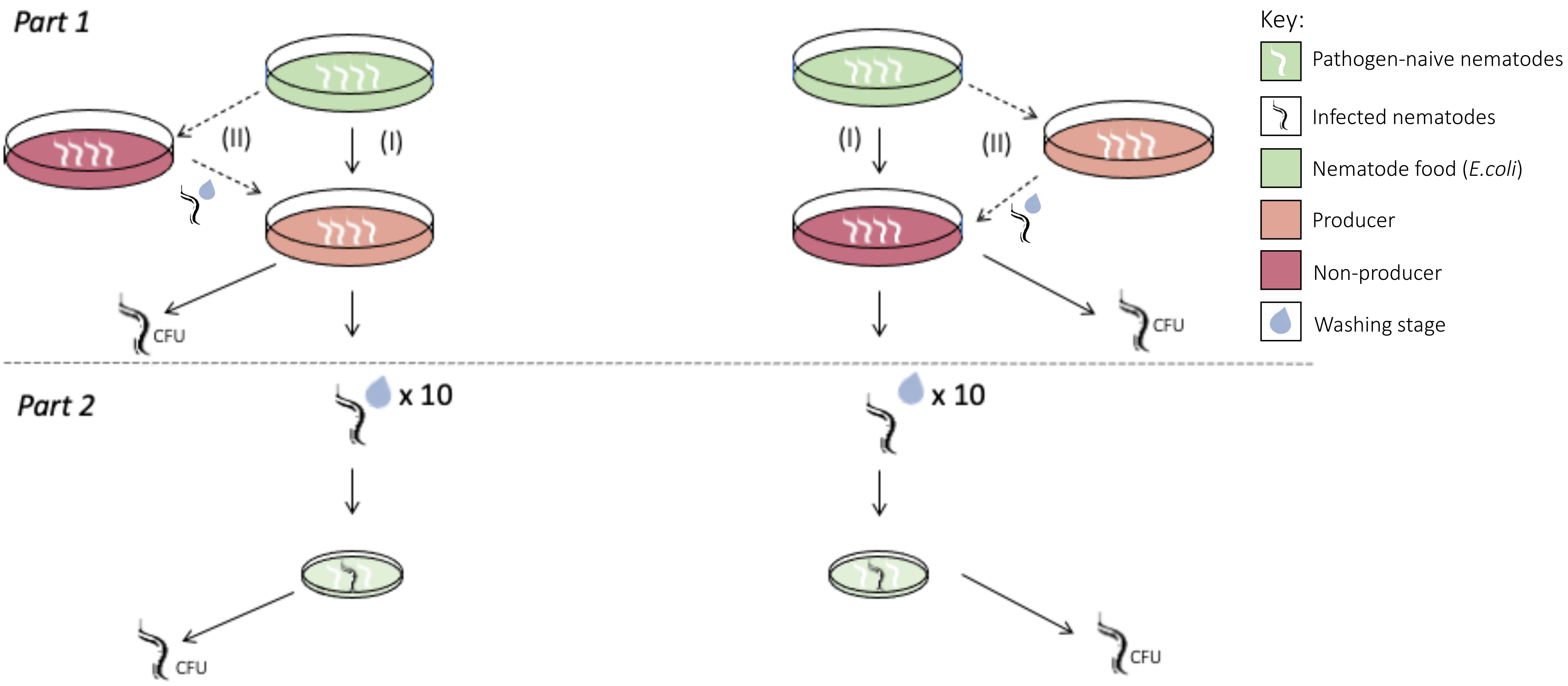

Supplementary Figure 1: Investigating within- and between-host dynamics. Within-host dynamics (I) Nematodes are grown up on food (OP50) until they reach fourth larval stage (L4) when they are exposed to either the producer or a non-producer (1 or 2) of *P. aeruginosa* for 24 hours. The nematodes are washed and the pathogen CFU's are calculated per worm. (II) Follows the same steps as (I) but with one added exposure stage and a transfer after 12 hours. Between-host dynamics: ten washed nematodes are transferred to a population of GFP-labelled uninfected worms for 24 hours to transmit infection. Pathogen CFU's per GFP-labelled nematode are recorded.

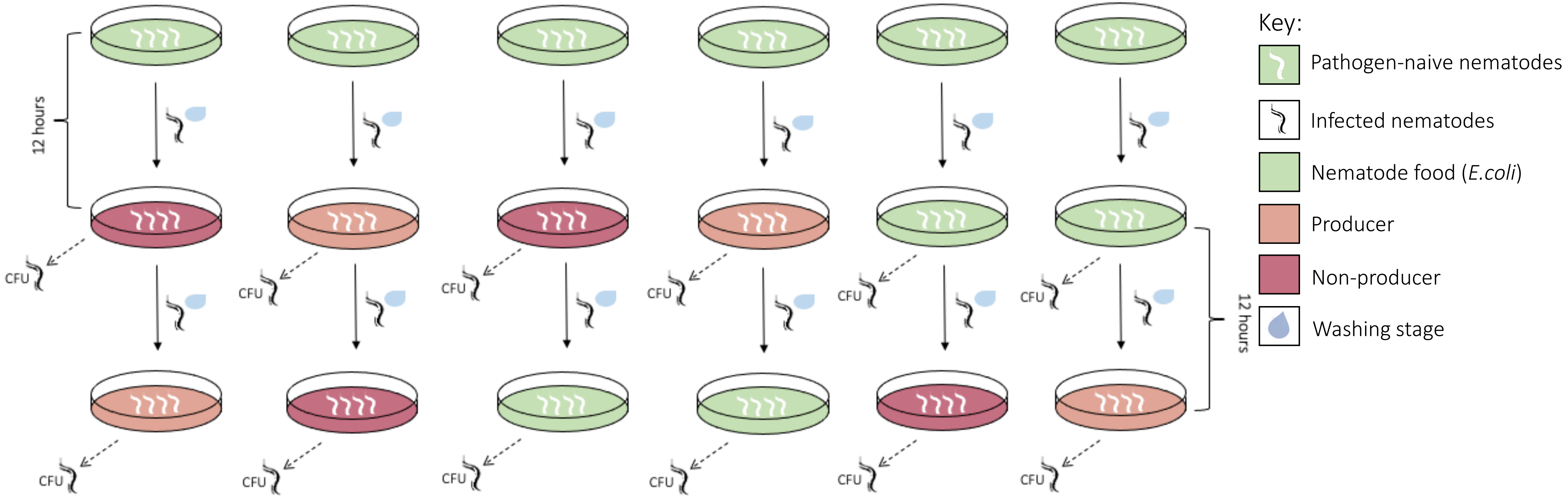

Supplementary Figure 2: Experimental set up for investigating within-host pathogen loads at the primary (12 hours) and secondary (24 hours) exposure stages. Nematodes are washed between transfers.

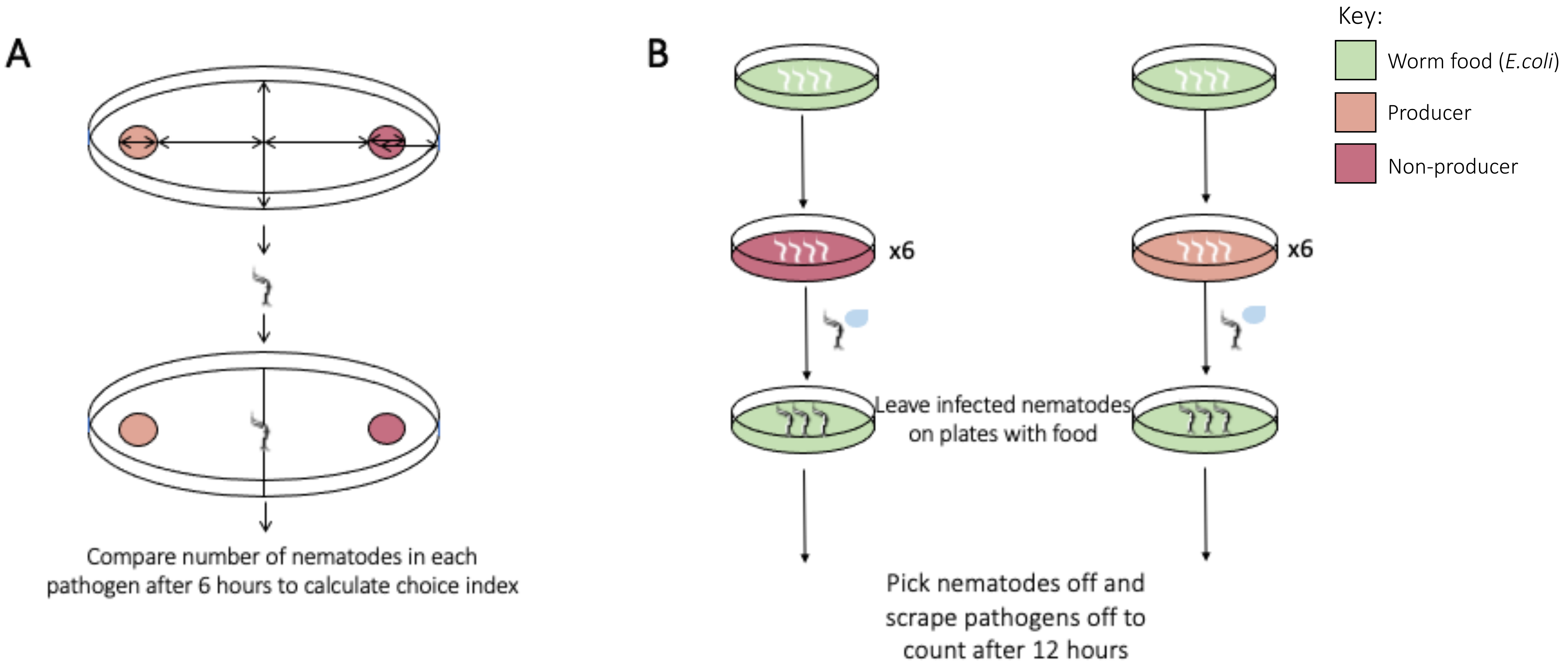

Supplementary Figure 3: Experiments investigating host preference for picking-up pathogens and host shedding of pathogens in the environment. A) Preference assay. Equal density samples of wild type producer (pale) and non-producer (dark) *P. aeruginosa* strains are placed equidistant from the center of the plate. 40 nematodes are added to the center of the plate and left for 6 hours at 20°C after which the number of nematodes in the bacteria are recorded to calculate a choice index. B) Host shedding of pathogens in the environment assay. Nematodes are exposed to a producer or non-producer for 24 hours, they are then washed and transferred to a new plate. After 12 hours the nematodes are picked off the plate and the CFUs grown from the plate.

Table S1: Within-host dynamics. Model output for single exposure and post-hoc test.

ANOVA output for transmission experiment stage 1 – non-producer only (exposed N2)

	df	Sum Sq	Mean Sq	F	P
Pathogen	2	1,396,773,445	698,386,723	39.797	1.52246987475705 x 10-11
Residuals	57	1,000,265,918	17,548,525		

Tukey post hoc test – non-producer only (exposed N2)

Comparison	Difference	CI Lower	CI Upper	Adjusted P
ΔLASR - PaO1	-11,201	-14,389	-8,014	4.74479344703127 x 10-11
ΔPVD - PaO1	-8,865	-12,053	-5,677	3.09603214176946x 10-8
ΔPVD - ΔLASR	2,337	-851	5,524	0.190944792332241

Table S2: Within-host dynamics A) Model output for two stage exposure and post hoc test output for non-producer CFU's and for B) producer CFU'S.

A) Likelihood ratio test for quasipoisson glm for non-producer CFU's

Model	Residual df	Residual Deviance	df	Deviance	P
Intercept only	79	139,611			
Pathogen	76	39,197	3	100,414	3.24176664162092 x 10-44

Tukey post hoc test for non-producer CFU's

Comparison	Difference	Std Error	Z	P
"PaO1→ΔPVD" - "PaO1→ΔLASR"	1.706	0.296	5.770	2.37994173257761 x10 -08
"ΔLASR→PaO1" - "PaO1→ΔLASR"	-0.646	0.464	-1.394	0.475890964188774
"ΔPVD→PaO1" - "PaO1→ΔLASR"	2.323	0.285	8.152	1.66533453693773 x 10-15
"ΔLASR→PaO1" - "PaO1→ΔPVD"	-2.352	0.393	-5.982	6.63580213000614x 10-09
"ΔPVD→PaO1" - "PaO1→ΔPVD"	0.617	0.144	4.293	0.000158475002713931
"ΔPVD→PaO1" - "ΔLASR→PaO1"	2.970	0.385	7.708	4.18554080283684x10-14

B) Likelihood ratio test for quasipoisson glm for producer CFU's

Model	Residual df	Residual Deviance	df	Deviance	P
Intercept only	79	55,976			
Pathogen	76	53,469	3	2,507	0.3648

Tukey post hoc test for producer CFU's

Comparison	Difference	Std Error	Z	P
"PaO1→ΔPVD" - "PaO1→ΔLASR"	-0.013	0.105	-0.122	0.999
"ΔLASR→PaO1" - "PaO1→ΔLASR"	-0.105	0.108	-0.971	0.766
"ΔPVD→PaO1" - "PaO1→ΔLASR"	0.083	0.103	0.808	0.851
"ΔLASR→PaO1" - "PaO1→ΔPVD"	-0.092	0.108	-0.848	0.831
"ΔPVD→PaO1" - "PaO1→ΔPVD"	0.096	0.103	0.930	0.789
"ΔPVD→PaO1" - "ΔLASR→PaO1"	0.188	0.106	1.775	0.285

Table S3A: Investigating interactions between pathogen and stage of exposure in within-host dynamics for A) Non-producer 2, B) Non-producer 1, C) Producer and D) Producer with the outlier removed.

A) ANOVA output - N2 12 and 24hr interaction – Non-producer ΔPVD

Term	Sum Sq	Mean Sq	Numerator df	Denominator df	F	P
Pathogen	777,454	777,454	1	16	0.654	0.430710191400823
Transfer	48,186,736	48,186,736	1	16	40.506	9.38616540965786e-06
Pathogen:Transfer	5,075,008	5,075,008	1	16	4.266	0.0554736896615176

B) ANOVA output - N2 12 and 24hr interaction - Non-producer ΔLAS

Term	Sum Sq	Mean Sq	Numerator df	Denominator df	F	P
Pathogen	6,236,119	6,236,119	1	32	4.525	0.0412
Transfer	5,561,736	5,561,736	1	32	4.036	0.0531
Pathogen:Transfer	8,817,600	8,817,600	1	32	6.398	0.0165

C) ANOVA output - N2 12 and 24hr interaction - Producer PaO1

Term	Sum Sq	Mean Sq	Numerator df	Denominator df	F	P
Pathogen	1,443,591	721,795	2	48	0.110	0.8960
Transfer	17,226,858	17,226,858	1	48	2.628	0.1116
Pathogen:Transfer	35,389,290	17,694,645	2	48	2.699	0.0775

D) ANOVA output - N2 12 and 24hr interaction - Producer PaO1 (outlier removed)

Term	Sum Sq	Mean Sq	Numerator df	Denominator df	F	P
Pathogen	8,044,702	4,022,351	2	46	0.879	0.4219
Transfer	32,474,175	32,474,175	1	46	7.100	0.0106
Pathogen:Transfer	38,804,516	19,402,258	2	46	4.242	0.0204

Table S3B: Differences between pathogen treatments when investigating the interactions between bacteria and stage of exposure in within-host dynamics for A) Non-producer 2, B) Non-producer 1 and C) Producer.

A) Primary (12hr) and secondary (24 hr) exposure differences – Non-producer ΔPVD

	Difference	Std Error	t	P
ΔPVD→PaO1	-3,065	514	-5.961	1.99478141444201e-05
ΔPVD→OP50	-1,563	514	-3.040	0.0078

B) Primary (12hr) and secondary (24 hr) exposure differences – Non-producer ΔLAS

	Difference	Std Error	t	P
ΔLAS→PaO1	-1,776	602	-2.952	0.0094
ΔLAS→OP50	204	602	0.339	0.7393

C) Primary (12hr) and secondary (24 hr) exposure differences - Producer PaO1

	Difference	Std Error	t	P
PaO1→ΔPVD	-980	1,302	-0.753	0.4590
PaO1→ΔLAS	-3,183	1,302	-2.445	0.0222
PaO1→OP50	774	1,302	0.595	0.5576

Table S4: Model output for comparing control CFU's when exposed to food to those exposed to a producer or non-producer treatment.

12 and 24 hr – secondary exposure stage – comparison of pathogen and food treatments

Second Pathogen	Term	df	Sum Sq	Mean Sq	F	P
ΔPVD	Pathogen	1	1,463,951	1,463,951	0.296	0.5941
	Residuals	16	79,201,173	4,950,073		
ΔLASR	Pathogen	1	12,500,000	12,500,000	18.525	0.0005
	Residuals	16	10,796,111	674,757		
PaO1	Pathogen	2	3,439,588	1,719,794	0.408	0.6697
	Residuals	24	101,241,975	4,218,416		

Table S5: Between host dynamics. Model output for single exposure stage and post-hoc tests.

ANOVA output for transmission experiment stage 1 – Non-producer only (exposed N2 Naive)

	df	Sum Sq	Mean Sq	F	P
Pathogen	2	266,000,423	133,000,212	7.726	0.0011
Residuals	57	981,175,992	17,213,614		

Tukey post hoc test – Non-producer only (exposed N2 Naive)

Comparison	Difference	CI Lower	CI Upper	Adjusted P
PaO1 - ΔLASR	3,440	283	6,597	0.0297
ΔPVD - ΔLASR	-1,608	-4,765	1,550	0.4433
ΔPVD - PaO1	-5,048	-8,205	-1,891	0.0009

Table S6: Between host dynamics. Model output for two stage exposure and post-hoc tests for A) non-producers and B) producer.

A) Likelihood ratio test for quasipoisson glm non-producers (exposed N2 naive)

Model	Residual df	Residual Deviance	df	Deviance	P
Intercept only	79	133,436			
Pathogen	76	39,259	3	94,178	9.79059749265308e-34

Tukey post hoc test non-producers (exposed N2 naive)

Comparison	Difference	Std Error	Z	P
"PaO1→ΔPVD" - "PaO1→ΔLASR"	2.575	1.272	2.024	0.1543735260584
"ΔLASR→PaO1" - "PaO1→ΔLASR"	2.823	1.262	2.237	0.0957031056723139
"ΔPVD→PaO1" - "PaO1→ΔLASR"	4.857	1.231	3.946	0.000339384657785713
"ΔLASR→PaO1" - "PaO1→ΔPVD"	0.249	0.451	0.551	0.93841964903327
"ΔPVD→PaO1" - "PaO1→ΔPVD"	2.282	0.355	6.423	4.31572111381229e-10
"ΔPVD→PaO1" - "ΔLASR→PaO1"	2.033	0.318	6.398	5.01496844051985e-10

B) Likelihood ratio test for quasipoisson glm producers (exposed N2 naive)

Model	Residual df	Residual Deviance	df	Deviance	P
Intercept only	79	80,169			
Pathogen	76	69,056	3	11,113	0.0112

Tukey post hoc test producers (exposed N2 naive)

Comparison	Difference	Std Error	Z	P
"PaO1→ΔPVD" - "PaO1→ΔLASR"	-0.065	0.136	-0.476	0.964243999944474
"ΔLASR→PaO1" - "PaO1→ΔLASR"	0.293	0.125	2.343	0.0884608791614553
"ΔPVD→PaO1" - "PaO1→ΔLASR"	0.222	0.127	1.747	0.298710576573992
"ΔLASR→PaO1" - "PaO1→ΔPVD"	0.358	0.128	2.808	0.0258013574782604
"ΔPVD→PaO1" - "PaO1→ΔPVD"	0.287	0.130	2.216	0.118429799065706
"ΔPVD→PaO1" - "ΔLASR→PaO1"	-0.071	0.118	-0.604	0.930652962888251

Table S7: Investigating host preference and the shedding of pathogens into the environment. A) Output for choice assay t-test and B) Output for shedding into environment assay and associated post-hoc test.

A) Choice assay - Producer vs Non-producer t-tests - output table

Comparison	Difference	Std Error	t	P
PaO1 - ΔLASR	0.047	0.061	0.773	0.445980843262594
PaO1 - ΔPVD	-0.033	0.049	-0.683	0.499995772345799
PaO1 - CHA0 Producer	0.006	0.034	0.188	0.853084623174605
PaO1 - CHA0 Non-producer	-0.405	0.033	-12.276	9.0882955154233e-11

B) Likelihood ratio test for quasipoisson glm – Pathogens shed into the environment (Lawn CFUs)

Model	Residual df	Residual Deviance	df	Deviance	P
Intercept only	17	81,119			
Pathogen	15	17,029	2	64,090	1.30607652055112e-13

Tukey post hoc test - Pathogens shed into the environment (Lawn CFUs)

Comparison	Difference	Std Error	Z	P
ΔLASR - PaO1	-0.825	0.112	-7.372	2.42361686275672e-13
ΔPVD - PaO1	-0.284	0.094	-3.014	0.00699912031736816
ΔPVD - ΔLASR	0.541	0.117	4.608	9.06211326889839e-06

Supplementary Methods - *Time Control Analysis*

With a two-pathogen treatment, within-host pathogen load was measured at the secondary exposure stage (24 hours). To investigate within-host pathogen load at the primary exposure stage (12 hours) we conducted an additional assay with an added stage of CFU collection after the primary exposure. We added a time control of nematode food OP50 to this assay (Figure S2). After 12 hours, CFUs were collected from 4-5 clean nematodes, and the remaining nematodes were washed and transferred to the secondary exposure stage (Figure S2). After 12 hours on the second plate, CFUs were collected from 4-5 clean nematodes. This experiment was replicated three times.

We made comparisons between two treatments for each of the three different pathogens: 1) primary exposure to pathogen (CFUs collected at 12 hours); and 2) nematode on lawn of food (OP50) for 12 hours and then secondary exposure to pathogen (CFUs collected at 24 hours). We used t-tests to compare mean CFUs per nematode between treatment 1 and treatment 2 for each of the three different pathogens (Figure S4). There was no significant difference between mean CFUs per nematode for treatment 1 and treatment 2 for any of the pathogens (Producer: $t = -0.616$, $p = 0.547$; Non-producer A: $t = -0.469$; $p = 0.646$; Non-producer B: $t = -0.462$, $p = 0.650$).

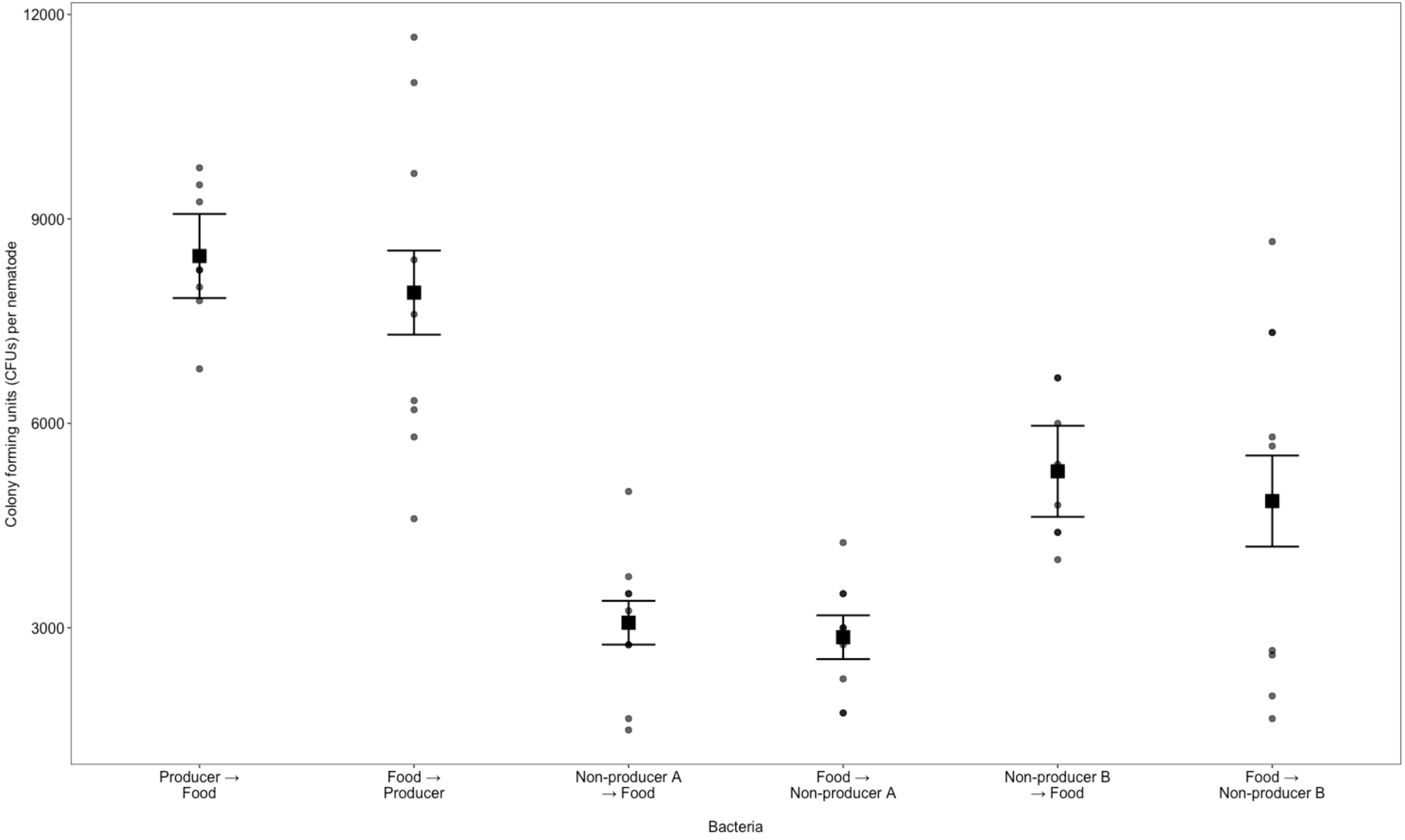

Figure S4. Time control analysis. The graph shows CFUs per nematode after 12 hours of exposure for each of the three different pathogens under two treatments: 1) primary exposure to pathogen (CFUs collected at 12 hours); and 2) nematode on lawn of food (OP50) for 12 hours and then secondary exposure to pathogen (CFUs collected at 24 hours). Across the three different pathogens, there were no significant differences between mean CFUs per nematode for treatment 1 and treatment 2. Square points with error bars represent mean +/- 1 SE. (Results of t-tests comparing treatments 1 and 2 for each pathogen – Producer: $t = -0.616$, $p = 0.547$; Non-producer A: $t = -0.469$, $p = 0.646$; Non-producer B: $t =-0.462$; $p = 0.650$).
